# Difference in the Vitreal Protein Profiles of Patients with Proliferative Diabetic Retinopathy with and without Intravitreal Conbercept Injection

**DOI:** 10.1155/2018/7397610

**Published:** 2018-04-19

**Authors:** Chen Zou, Minjie Zhao, Jingjing Yu, Dandan Zhu, Yunzhi Wang, Xinping She, Yanan Hu, Zhi Zheng

**Affiliations:** ^1^Department of Ophthalmology, Shanghai First People's Hospital, School of Medicine, Shanghai Jiao Tong University, No. 100 Haining Road, Shanghai 200080, China; ^2^Department of Ophthalmology, Yixing People's Hospital, Jiangsu University, No. 75 Tongzhenguan Road, Yixing, Jiangsu 214200, China; ^3^Department of Ophthalmology, Changshu the 2nd People's Hospital, Changshu, Jiangsu 215500, China

## Abstract

**Purpose:**

To examine the difference in the vitreal protein profiles of patients with proliferative diabetic retinopathy (PDR) with and without preoperative intravitreal conbercept (IVC) treatment.

**Methods:**

Liquid chromatography-tandem mass spectrometry- (LC-MS/MS-) based proteomic methods were used to determine the protein profiles of the vitreous humor in patients with PDR treated with (IVC group; *n* = 9) and without (PDR group; *n* = 8) preoperative IVC. Gene ontology (GO) annotation and REACTOME pathway analysis were obtained to overview differentially expressed proteins between each group. Intravitreal levels of apolipoprotein A-II (APOA2) and ceruloplasmin (CP) were measured using enzyme-linked immunosorbent assays.

**Results:**

307 proteins were expressed differentially between PDR and IVC groups, including 218 proteins downregulated in response to IVC. The most notable GO annotations in level 3 and REACTOME pathways describing the differentially expressed proteins were “innate immune response” and “platelet degranulation.” The intravitreal levels of APOA2 and CP were lower in the IVC group than in the PDR group (*p* < 0.01).

**Conclusions:**

In addition to decreasing the intravitreal vascular endothelial growth factor level, IVC may alter the vitreal protein profile in patients with PDR, with the differentially regulated proteins involved in the immune response, platelet degranulation, complement activation, and inflammation.

## 1. Introduction

Vascular endothelial growth factor (VEGF) is the major mediator of intraocular neovascularization and abnormal vessel permeability and plays a pivotal role in the pathogenesis of diabetic retinopathy [1, 2]. Therefore, VEGF has become a key target in antiangiogenic therapy [3]. Several drugs that act against VEGF have been developed; these drugs include bevacizumab (Avastin; Novartis), a recombinant humanized monoclonal antibody, and ranibizumab (Lucentis; Novartis), a Fab fragment that is similar to bevacizumab. In comparison, VEGF Trap (aflibercept; Regeneron Pharmaceuticals) is a novel soluble decoy receptor generated with Trap technology that uses fused components from multiple endogenous receptors [4]. Similarly, conbercept (Kanghong Biotechnologies) is a recombinant fusion protein composed of the second immunoglobulin (Ig) domain of vascular endothelial growth factor receptor-1 (VEGFR1) and the third and fourth Ig domains of vascular endothelial growth factor receptor-2 (VEGFR2), fused to the constant region (Fc) of human IgG1, and it acts as a receptor with affinity for all informs of VEGF and placental growth factor (PlGF) [5–7].

Intravitreal injection of aflibercept is used widely in the treatment of proliferative diabetic retinopathy (PDR). Patients with PDR who are treated with intravitreal aflibercept have better outcomes than those treated with panretinal photocoagulation [8, 9]. Moreover, preoperative intravitreal aflibercept decreased the rates of intra- and postoperative bleeding during vitrectomy and improved the best-corrected visual acuity (BCVA) in patients with PDR [10]. Similarly, intravitreal conbercept (IVC) is better than posterior subtenon triamcinolone acetonide for diabetic macular edema in the proliferative stage of PDR [11]. Preoperative conbercept treatments also allow patients with PDR to undergo surgery more quickly, with less risk of intra- or postoperative bleeding, and result in a better postoperative BCVA [12, 13]. Moreover, conbercept and ranibizumab are effective in the treatment of diabetic macular edema, with similar clinical efficacy, but conbercept has a longer treatment interval and fewer IVC injections are needed [14]. A previous proteomics study found that IVC alters the protein profile in the rabbit retina. However, whether IVC alters the protein profile in humans with PDR is not clear, and the mechanisms of the effects of IVC treatment on PDR are not fully understood. Hence, exploration of the mechanism underlying the actions of anti-VEGF treatment agents on PDR is important.

In this study, we used a proteomics method to compare the protein profiles of vitreous humor from patients with PDR who were treated and not treated with IVC. We identified 307 proteins in vitreous humor that were expressed differentially in untreated patients with PDR and those who were treated with IVC. The IVC treatment not only decreased the intravitreal VEGF level in patients with PDR but also altered the levels of proteins regulating processes such as inflammation, apoptosis, angiogenesis, immune responses, bleeding, and coagulation. Therefore, apolipoprotein A-II (APOA2) and ceruloplasmin (CP) may be involved in the development of PDR and in the mechanism of the effects of anti-VEGF treatment.

## 2. Materials and Methods

### 2.1. Patients and Sample Collection

Diabetic patients were included if they had PDR-related complications, including persistent vitreous hemorrhage of more than 1-month duration in a patient with no history of panretinal photocoagulation (PRP), nonclearing vitreous hemorrhage in a patient with a history of complete PRP, vitreous hemorrhage with retinal detachment by B-scan ultrasonography, or macula-involving or macula-threatening tractional retinal detachment (TRD). The protocol was approved by the hospital's research ethics committee. Informed consent was obtained from each patient, and the experimental procedures followed the tenets of the Declaration of Helsinki. Patients' rights to privacy were protected in this study. All patients underwent 25G pars plana vitrectomy (PPV). PDR patients were randomly assigned to one of two groups. One group was preoperatively treated with IVC (*n* = 9). Intravitreal injection of conbercept (1.0 mg) was performed 5–7 days before PPV. Those not treated with IVC were defined as the PDR group (*n* = 8). Nondiabetic patients with idiopathic macular hole (iMH) made up the control group (*n* = 9). Criteria for exclusion were history of photocoagulation treatment, history of ocular trauma or surgery, vasculopathy other than PDR (including retinal vein obstruction or retinal vasculitis), other ocular disease (including neovascular glaucoma, age-related macular degeneration, or rhegmatogenous retinal detachment), and systemic disease other than diabetes mellitus (including autoimmune disease or high blood pressure). The fasting blood glucose (FBG) levels of all patients were determined preoperatively, while glycosylated hemoglobin (HbA1C) was determined for the PDR and IVC group patients but not for MH patients, to avoid overexamination. During PPV, a 0.2–0.3 mL sample of undiluted vitreous humor was removed from the posterior segment using a 25G vitreous cutter and centrifuged at 15000 rpm for 10 min at 4°C. The supernatant was stored under liquid nitrogen for later analysis.

### 2.2. Sample Preparation

Protein content was measured using a bicinchoninic acid (BCA) kit (Beyotime Biotechnology, Jiangsu, China) according to the manufacturer's instructions. To 1 volume of cold sample, 1/3 volume of 100% (*w*/*v*) TCA (6.1 N, Sigma) was added and mixed well to give a final TCA concentration of 20–25%. The solution was held on ice for 4 h in a cold room (4°C) then centrifuged for 30 min at 4°C. The supernatant was aspirated with a gel loading tip, leaving 5–10 *μ*L in the tube so as not to disturb the pellet, which was then washed twice with 500 *μ*L ice-cold acetone. After each wash, the sample was centrifuged for 10 min and dried, using either a speed vacuum or room air for 1-2 min. The washed protein pellet was dissolved in 8 M urea with 100 mM Tris-HCl at pH 8.5. To reduce disulfide bonds, tris (2-carboxyethyl) phosphine (TCEP) was added (to a final concentration, of 5 mM) and incubated for 20 min at room temperature. The residue was alkylated using iodoacetamide (final concentration, 10 mM) for 15 min at room temperature. The protein mixture was diluted four folds and digested with trypsin (Promega) added 1 : 100 *w*/*w*.

### 2.3. LC-MS/MS Analysis and Data Analysis

Liquid chromatography-tandem mass spectrometry (LC-MS/MS) analysis was performed at the National Center for Protein Science, Shanghai, as previously described [15, 16]. (See supplementary methods for a description of this procedure.) Normalized spectral abundance factor (NSAF) which was firstly proposed by Florens et al. [17] was used to evaluate the relative protein contents, based on the spectrum counts. This method uses protein length to normalize spectral count (SC) for improving the accuracy. For each protein in a given database, the NSAF score is NSAF_*N*_ = SN/LN/∑_*i*=1_^*n*^ *si*/*Li*, where *N* is protein index; SN is the number of peptide spectra matched to the protein; LN is the length of protein *N*; *n* is the total number of proteins in the input database. In Reference to the method reported by Wiśniewski et al. [16], quantifiable proteins in the analysis of samples were defined as those identified at least 50% in at least one type of sample. STRAP 1.5 and DAVID Bioinformatics Resources 6.8 (https://david.ncifcrf.gov/tools.jsp) were used to obtain gene ontology (GO) annotation and REACTOME pathway analysis. STRING 10.5 (https://string-db.org/cgi/input.pl) was used to obtain interaction network.

### 2.4. Validation of Proteomic Analysis

To confirm changes in the intravitreal level of specific proteins, enzyme-linked immunosorbent assay (ELISA) were performed using ELISA kits (CUSABIO) according to the manufacturer's instructions. Total protein content was calculated based on OD values and dilution ratios.

### 2.5. Statistical Methods

SPSS (version 17) and SAS (version 9) were used for statistical analysis. All continuous variables exhibited a typical normal distribution, as tested by the Shapiro-Wilk method. Comparisons among groups were conducted using one-way ANOVA. Multiple test correction was performed by SNK method. The level of statistical significance was *p* < 0.05.

## 3. Results

### 3.1. Baseline Findings

Table 1 summarizes the demographic and laboratory data. No significant difference was observed between groups, except in the fasting plasma glucose level (*p* < 0.01).

### 3.2. Differentially Expressed Proteins in Response to IVC Determined by LC-MS/MS

In total, 654 proteins were detected in the vitreous humor from the PDR group and 536 were detected in samples from the IVC group. Of these proteins, 204 were identified only in the PDR group (Supplementary Table S1) and 86 were identified only in the IVC group (Supplementary Table S2). Four hundred fifty proteins were common to both groups, and 586 proteins were identified in the control group. Among the 450 proteins identified in both groups, 3 proteins were significantly upregulated and 14 proteins were significantly downregulated in the IVC group compared with the PDR group (*p* < 0.05; Supplementary Table S3). Therefore, a total of 307 proteins was differentially expressed: 218 proteins, including 204 proteins identified only in the PDR group and 14 proteins downregulated in the IVC group, were downregulated in response to IVC, whereas 89 proteins, including 86 proteins identified only in the IVC group and 3 proteins upregulated in the IVC group, were upregulated in response to IVC.

### 3.3. Gene Ontology Annotation and Pathway Analysis of Proteins Expressed Differentially in Response to IVC

#### 3.3.1. Annotations and Pathways Associated with All Differentially Expressed Proteins

**Figure 1:**
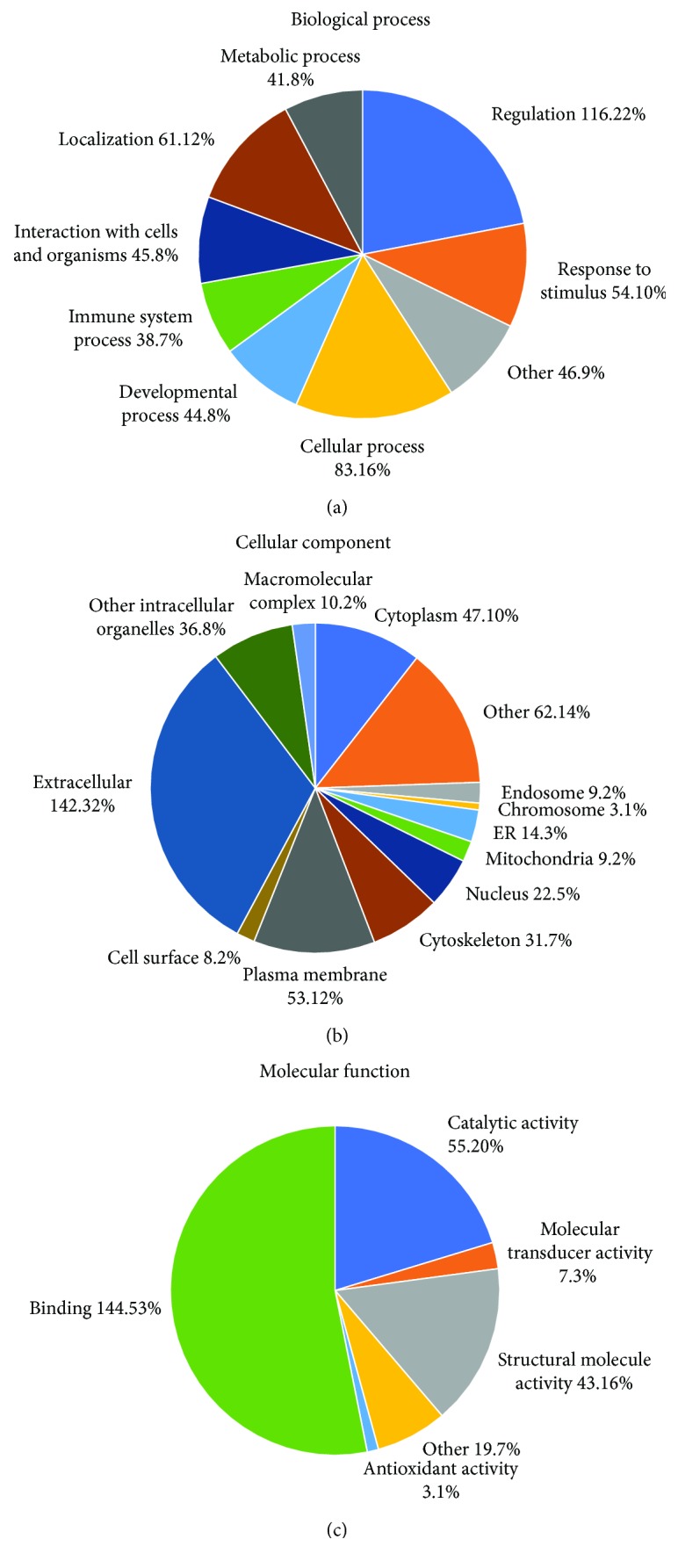
Annotations in level 1 of differentially expressed proteins in (a) biological process, (b) cellular component, and (c) molecular function category according to STRAP software.

To obtain a functional overview of the differentially expressed proteins identified by LC-MS/MS, gene ontology (GO) annotation and REACTOME pathway analysis were performed using STRAP Software and DAVID Bioinformatics Resources. Each protein was assigned to three categories including biological processes, molecular functioning, and cellular components. Single protein could be annotated to several subcategories in level 1 to level 3 redundantly.  Figure 1 listed the annotations in level 1 in the “biological process,” “cellular component,” and “molecular function” category according to STRAP software. The proteins were further classified into different subcategories in level 1 (Figure 1). The highest number of proteins in each category were involved in “regulation” (116 proteins, Figure 1(a)), “extracellular” (142 proteins, Figure 1(b)), and “binding” (144 proteins, Figure 1(c)).

**Figure 2:**
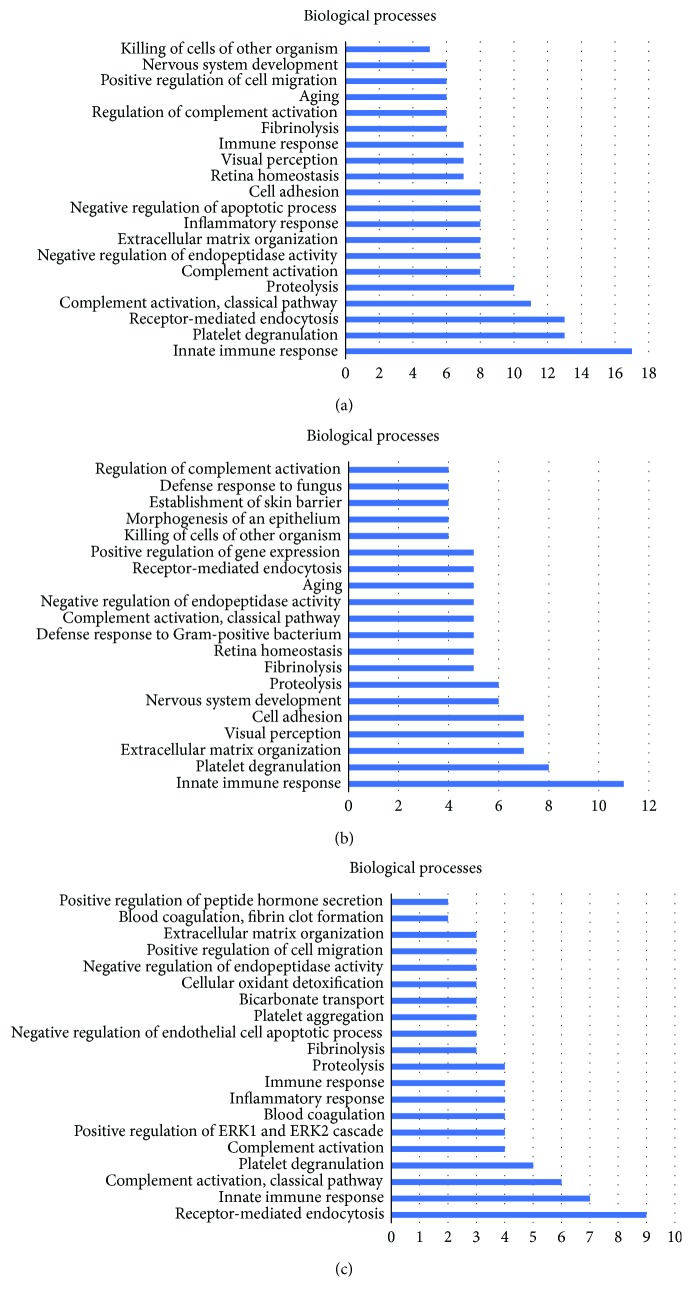
GO annotation of the main altered proteins in response to IVC. (a) The most notable 20 of 111 GO annotations of all the differentially expressed proteins. (b) The most notable 20 of 87 GO annotations of the decreased proteins. (c) The most notable 20 of 42 GO annotations of the increased proteins.

**Figure 3:**
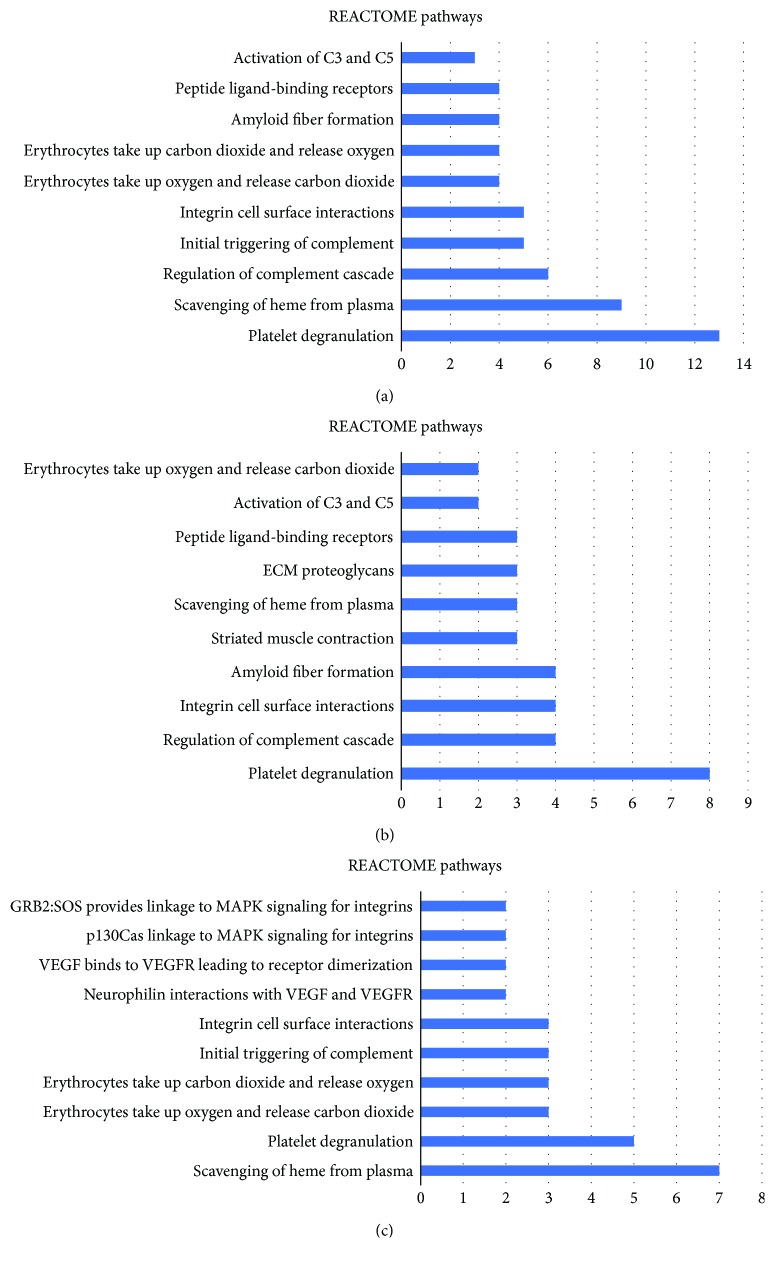
REACTOME pathway analysis of the altered proteins in response to IVC. (a) The most notable 10 of 19 REACTOME pathways of all the differentially expressed proteins. (b) The most notable 10 of 14 REACTOME pathways of the decreased proteins. (c) The most notable 10 of 15 REACTOME pathways of the increased proteins.

Moreover, 81 downregulated proteins and 27 upregulated proteins were searched in DAVID Bioinformatics Resources. These differentially expressed proteins were annotated to 197 annotations in level 3 and associated with 19 REACTOME pathways.  Figure 2(a) shows the most notable 20 of the 111 GO annotations in level 3 in the “biological process” category, with the most common being “innate immune response” (17 proteins), followed by “platelet degranulation” (13 proteins) and “receptor-mediated endocytosis” (13 proteins). In addition, 41 annotations in level 3 were in the “molecular function” category, with the most notable being “protein binding” (59 proteins). Forty-five annotations in level 3 were in the “cellular component” category, with “extracellular exosome” (69 proteins) being the most notable.  Figure 3(a) shows the most notable 10 of 19 REACTOME pathways, with the most common being “platelet degranulation” (13 proteins), followed by “scavenging of heme from plasma” (9 proteins) and “regulation of complement cascade” (6 proteins).

#### 3.3.2. Annotations and Pathways Associated with Proteins Decreased in Response to IVC

The proteins that decreased in response to IVC involved 150 GO annotations in level 3 and 14 REACTOME pathways. The most notable 20 of 87 GO annotations in level 3 involved in the “biological process” category were “innate immune response” (11 proteins), followed by “platelet degranulation” (8 proteins) and “extracellular matrix organization” (7 proteins; Figure 2(b)). Twenty-six annotations in level 3 were involved in the “molecular function” category, with the most notable being “protein binding” (44 proteins). Thirty-seven annotations in level 3 were in the “cellular component” category, with the most notable being “extracellular exosome” (53 proteins).  Figure 3(b) shows the most notable 10 of 14 REACTOME pathways, with the most common being “platelet degranulation” (8 proteins), followed by “regulation of complement cascade” (4 proteins), “integrin cell surface interactions” (4 proteins), and “amyloid fiber formation” (4 proteins).

#### 3.3.3. Annotations and Pathways Associated with Proteins Increased in Response to IVC

The proteins that increased in response to IVC were represented by 70 GO annotations in level 3 and 15 REACTOME pathways. The most notable 20 of 42 GO annotations in level 3 involved in the “biological process” category included “receptor-mediated endocytosis” (9 proteins), followed by “innate immune response” (7 proteins) and “complement activation” (6 proteins; Figure 2(c)). Fifteen annotations in level 3 were involved in the “molecular function” category, with the most notable being “protein binding” (18 proteins). Thirteen annotations in level 3 were in the “cellular component” category, with the most notable being “extracellular region” (20 proteins).  Figure 3(c) shows the most notable 10 of 15 REACTOME pathways, with the most common being “scavenging of heme from plasma” (7 proteins), followed by “platelet degranulation” (5 proteins).

### 3.4. Interaction Networks

**Figure 4:**
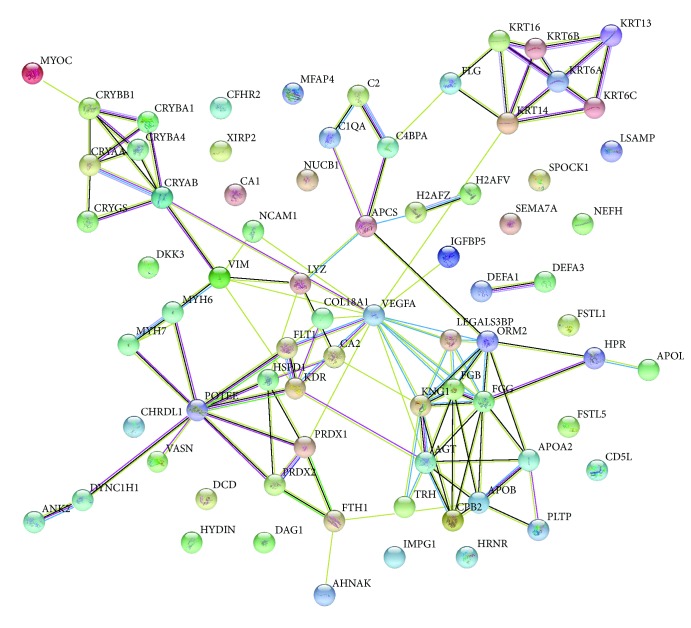
Interaction networks of differentially expressed proteins in response to IVC according to STRING tools.

By using STRING tools, we found that the differentially expressed proteins in response to IVC exist in interaction networks. The direct interaction network is shown in Figure 4. There were 74 nodes and 114 edges in this network. The most notable 10 nodes were VEGFA, KDR, FLT1, FGG, FGB, APOB, APOA2, KNG1, AGT, and ORM2.

### 3.5. Quantitative Validation of Candidate Proteins by ELISA

**Figure 5:**
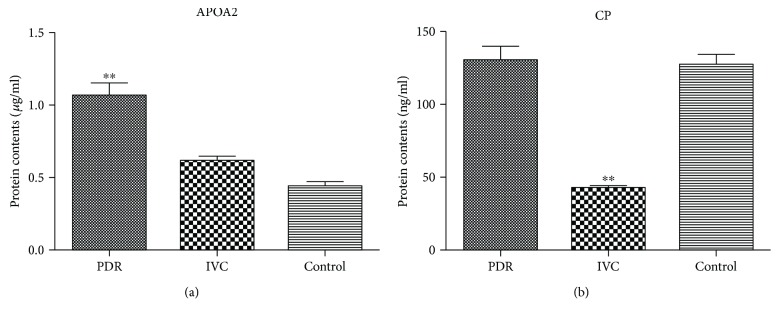
Intravitreal level of (a) APOA2 and (b) ceruloplasmin measured by ELISA. ∗∗= statistically different with other groups (*p* < 0.01).

We focused on candidate proteins that were related to inflammation, apoptosis, angiogenesis, fibrosis, and tumors and are seldom studied in diabetic retinopathy. WE screened for APOA2 and ceruloplasmin CP were screened. We performed an enzyme-linked immunosorbent assay (ELISA) analysis to confirm the changes in the intravitreal levels of the candidate proteins. The intravitreal APOA2 level was significantly higher in the PDR group than in controls (*p* < 0.01) and was decreased in response to IVC (*p* < 0.01; Figure 5). The CP level did not differ between the PDR and control groups (*p* > 0.05) and was significantly decreased in the IVC group (*p* < 0.01). The intravitreal changes measured by ELISA were generally consistent with the LC-MS/MS results.

## 4. Discussion

Previously, we found that preoperative intravitreal ranibizumab injection in patients with severe PDR contributed to a decreased risk of postoperative neovascular glaucoma [18], which is among the most serious postoperative complications of PDR. Similar to aflibercept, conbercept (Kanghong Biotechnologies), the newest VEGF Trap, is a recombinant fusion protein composed of the second Ig domain of VEGFR1 and the third and fourth Ig domains of VEGFR2 fused to the constant region of human IgG1, and it acts as a receptor with affinity to all informs of VEGF and PlGF [5–7]. Preoperative conbercept treatment also allows patients with PDR to undergo surgery more quickly, with less risk of intra- or postoperative bleeding, and to have better postoperative BCVA [12, 13], but the mechanisms are not fully understood.

Recently, Wang et al. [19] quantified 57 downregulated and 50 upregulated proteins after IVC treatment in rabbit retinas using tandem mass tag quantitative mass spectrometry. Differentially expressed proteins were enriched in 47 Kyoto Encyclopedia of Genes and Genomes pathways, including “platelet activation,” “measles,” and “complement coagulation cascades.” However, the authors found no significant changes in proteins associated with inflammation or apoptosis in the conbercept-injected eyes. There are two potential reasons that may explain the difference on the results between Wang et al. and our current study. First, they used vitreous humor sample from rabbit instead of human. Second, they did not create a diabetic retinopathy model. While our vitreous humor samples were obtained from human patients of diabetic retinopathy, the current study is the first to examine the effects of conbercept, the newest anti-VEGF agent, via evaluation of vitreous humor from patients with PDR.

We identified 307 proteins, including 218 downregulated proteins and 89 upregulated proteins that were expressed differentially in response to IVC. We first submitted all of these proteins to STRAP software to overview the total differentially expressed proteins by classifying it to three categories including “biological process,” “cellular component,” and “molecular function.” The highest number of proteins in each category were involved in “regulation”, “extracellular,” and “bingding.” However, these annotations were in level 1, which cannot provide more specific information about the location and function of these differentially expressed proteins. Therefore, we further submitted these proteins to DAVID Bioinformatics Resources to obtain annotations in level 3 and REACTOME pathways. We found that these differentially expressed proteins play roles mainly in the innate immune response, platelet degranulation, endocytosis, scavenging of heme from plasma, and regulation of the complement cascade, suggesting that the effects on VEGF involve these signaling pathways. Therefore, the therapeutic effects of IVC in PDR involve not only VEGF reduction but also signaling pathway regulation. Moreover, we found that the differentially expressed proteins in response to IVC exist in interaction networks. The key nodes include VEGFA, KDR, and FLT1, which indicated that these proteins may have a critical role in PDR and the mechanisms of the effect of IVC.

Next, we separately submitted the upregulated and downregulated proteins to the database and found that “platelet degranulation” and “innate immune response” mainly described the proteins that were decreased, but not those that were increased, in response to IVC.

During degranulation at a site of injury, platelets release several polypeptides and small molecules that supplement thrombin generation, such as fibrinogen, growth factors, and protease inhibitors. Diabetes can alter the homeostasis between platelet aggregation and degranulation, which protects against vascular damage. IVC treatment has been suggested to regulate blood coagulation and vascular damage in PDR by inhibiting a series of proteins involved in platelet degranulation.

The immune response plays critical roles in diabetic retinopathy via the presence of antipericyte and antiendothelial cell autoantibodies and the abnormal expression of T cells [20–22]. Moreover, VEGF enhances the innate immune response by respiratory epithelial cells [23]. Our data confirmed the association between VEGF and the immune response. The results suggest that conbercept treatment regulates the immune response by inhibiting VEGF, affecting the development of diabetic retinopathy.

Nevertheless, although many differentially expressed proteins were involved in complement regulation, we found that the numbers of decreased and increased proteins involved in complement regulation were similar. Complement cascade activation is regulated by a family of related proteins called the regulators of complement activation (RCA). RCA deposition and the complement system have important roles in tissue homeostasis, clearing dead cells and debris and preventing damage from oxidative stress [24]. A previous proteomics study showed that many proteins expressed differentially in response to diabetic retinopathy were involved in complement regulation. However, whether IVC has positive or negative regulatory effects on the complement system in PDR remains unclear. Further quantitative analyses of the main proteins involved in complement regulation in PDR are needed.

Of the differentially expressed proteins, we were particularly interested in APOA2 and CP, which play critical roles in lipoprotein metabolism, inflammation, oxidative stress, apoptosis, and other processes associated closely with the development of diabetic retinopathy. The ELISA results were generally consistent with those of LC-MS/MS, suggesting that these proteins are involved in the pathogenesis of diabetic retinopathy and the mechanism of the effect of IVC.

APOA2 is the second most abundant protein constituent of high-density lipoprotein (HDL) and is important in the metabolism of lipids and glucose [25, 26]. Studies of transgenic mice have suggested that APOA2 confers proinflammatory properties on HDL [27]. Moreover, APOA2 can augment the monocyte response to lipopolysaccharide (LPS) by suppressing the inhibitory activity of LPS-binding protein [28]. These findings suggest that APOA2 acts as a proinflammatory cytokine.

CP is an abundant, blue plasma protein that carries approximately 95% of the total circulating copper in adult humans. CP usually has antioxidant properties, but in conditions such as diabetes mellitus and hyperhomocysteinemia, it promotes vasculopathic effects that include lipid oxidation, the negation of nitric oxide bioactivity, and endothelial cell apoptosis via its copper content [29]. Moreover, copper stimulates blood vessel formation by promoting endothelial cell migration and angiogenesis [30, 31]. These findings suggest that CP is involved in the development of inflammation, apoptosis, and angiogenesis. Our data show that the intravitreal levels of APOA2 and CP were inhibited in the vitreous humor of patients with PDR in response to IVC, suggesting that these proteins are involved in the development of diabetic retinopathy and that the mechanism of the effects of anti-VEGF treatment occurs via their effects on inflammation, apoptosis, and/or angiogenesis.

However, there were some limitations in this study. First, we enrolled nondiabetic iMH patients as the control group. While the best option to the control group is normal eyes or donated eyes, we cannot get vitreous humor from normal people, which is not ethical, and donated eyes were hardly available. Secondly, the sample size of each group was small. We will enroll more patients for further research.

## 5. Conclusion

In summary, using proteomics methods, 307 proteins that were differentially expressed in the vitreous humor of patients with PDR who were and were not treated with IVC were identified. Preoperative IVC treatment may regulate not only the level of VEGF, but also those of proteins involved in the immune response, platelet degranulation, complement activation, inflammation, endocytosis, proteolysis, and heme scavenging in the vitreous humor of patients with PDR. Importantly, the APOA2 and CP levels were altered and these proteins may be involved in the pathogenesis of PDR and the mechanism of anti-VEGF treatment. These findings highlight new targets and pathways for the treatment of PDR and help to clarify the mechanism underlying the effects of anti-VEGF treatment in PDR.

## Figures and Tables

**Table 1:** Patient characteristics.

	Control (*n* = 9)	PDR (*n* = 8)	IVC (*n* = 9)	*p* value
Age (years)	53.9 ± 12.5	47.5 ± 10.7	50.9 ± 11.8	>0.05
Gender (male/female)	4 : 5	4 : 4	4 : 5	>0.05
FBG (mmol/L)	6.0 ± 0.7	9.7 ± 0.9	9.8 ± 2.0	<0.01
HbA1C (%)	—	9.1 ± 1.3	8.5 ± 1.2	>0.05
Lensstatus (phakic/pseudophakic)	3/6	3/5	4/5	>0.05
Indication for surgery				
VH	—	8 (100%)	9 (100%)	>0.05
TRD	—	2 (25%)	3 (33%)	>0.05

VH: vitreous hemorrhage; TRD: tractional retinal detachment. TRD: tractional retinal detachment. FBG: fasting blood glucose.

## Supplementary Materials

Supplementary Materials Supplementary methods: a further description of the methods of LC-MS/MS analysis. Supplementary Table S1: 204 proteins identified only in PDR group. Supplementary Table S2: 86 proteins identified only in IVC group. Supplementary Table S3: Differentially expressed proteins among the 450 protein detected in both PDR and IVC group.
