# Supplementary material for: Difference in the Vitreal Protein Profiles of Patients with Proliferative Diabetic Retinopathy with and without Intravitreal Conbercept Injection
Source: J Ophthalmol. 2018 Apr 19;2018:7397610. doi: 10.1155/2018/7397610 (PMC5932980; doi:10.1155/2018/7397610)
Supplement: Supplementary Materials — Supplementary methods: a further description of the methods of LC-MS/MS analysis. Supplementary Table S1: 204 proteins identified only in PDR group. Supplementary Table S2: 86 proteins identified only in IVC group. Supplementary Table S3: Differentially expressed proteins among the 450 protein detected in both PDR and IVC group. [file 7397610.f1.pdf]

Difference in the Vitreal Protein Profiles of Patients with Proliferative Diabetic Retinopathy with and without Intravitreal Conbercept Injection

Chen Zou[1], Minjie Zhao[2], Jingjing Yu[3], Dandan Zhu[1], Yunzhi Wang[1], Xinping She[1], Yanan Hu[1] & Zhi Zheng[1].

1. Department of Ophthalmology, Shanghai First People's Hospital, School of Medicine, Shanghai Jiao Tong University, No. 100 Haining Road, Shanghai 200080, China.

2. Department of Ophthalmology, Yixing People's Hospital, Jiangsu University, No.75 Tongzhenguan Road, Yixing, Jiangsu, 214200, China.

3. Department of Ophthalmology, Changshu the 2nd People's Hospital, Changshu, Jiangsu, 215500, China.

First author's E-mail: chenzou0814@foxmail.com

Corresponding author: Zhi Zheng. E-mail: zzheng88@sjtu.edu.cn

Supplementary methods

LC-MS/MS analysis

A C18 column (100 μm ID, 15 cm length) packed with Aqua C18 reverse phase material (Phenomenex) was placed inline with a PROXEON EASY-nLC1000 liquid chromatography system (ThermoFisher, San Jose, CA). Mobile phases A and B were 0.1% formic acid in water and 0.1% formic acid in acetonitrile, respectively. After loading, the digested peptides were separated using the following gradient: 0–5 min, 0–3% B; 5–150 min, 3–30% B; 150–166 min, 30–100% B; 166–180 min, 100% B. The flow rate was set to 300 nL/min. Peptides eluted from the LC column were directly electrosprayed into an Orbitrap Elite mass spectrometer (ThermoFisher, San Jose, CA) with the application of a distal 1.8 kV spray voltage. A cycle of 1 full-scan MS spectrum (m/z 300–1800) was acquired followed by 20 data-dependent MS scans, sequentially generated for the 20 most intense ions in the full MS spectrum at a 30% normalized collision energy. The number of microscans for both MS and MS/MS scans was one and the maximum ion injection times were 200 and 50 ms, respectively. The dynamic exclusion duration was 60 s. MS scan functions and HPLC solvent gradients were controlled by the Xcalibur data system (ThermoFisher).

Data Analysis

Protein analysis was performed using the Integrated Proteomics Pipeline, IP2 (Integrated Proteomics Applications, Inc., San Diego, CA). MS/MS spectra

were extracted from raw files using RawXtract 1.9.9.1 (http://fields.scripps.edu/downloads.php), and were searched with the ProLuCID algorithm against the Uniprot human database (http://www.uniprot.org/) within IP2 (MS1: 7ppm, MS2: 0.05 Da). To accurately estimate peptide probabilities and false discovery rates, we used a decoy database containing the reverse sequences of all proteins appended to the target database. The search space included all fully tryptic and half-tryptic peptide candidates that fell within the mass tolerance window. Carbamidomethylation (+57.02146 Da) of cysteine was considered a static modification. The database search results were assembled and filtered using the DTASelect program. Normalized spectral abundance factor (NSAF) was used to evaluate the relative protein contents base on the spectrum counts. The protein detected in less than 50% of the sample was defined to undetected protein.

supplementary table S1: 204 proteins identified only in PDR group

**Uniprot ID**	**sequence coverage(%)**	**sequence count**	**n**[a]	**mean NSAF**[b]
A0A024QZ34	12.65	2.50	4	0.00014
A0A024R2W4	5.11	2.80	4	0.00011
A0A024R3X4	5.44	2.00	6	0.00011
A0A024R433	15.68	2.82	6	0.00060
A0A024RDK2	1.25	2.00	4	0.00001
A0A087WTE4	6.40	3.55	4	0.00006
A0A087WTF6	5.76	3.55	4	0.00006
A0A087WU91	17.50	2.00	4	0.00070
A0A087WV75	7.49	5.31	4	0.00005
A0A087WWD4	7.49	5.31	4	0.00005
A0A087WX77	7.71	5.31	4	0.00006
A0A097PIG2	32.76	7.40	4	0.00701
A0A097PIJ4	32.76	7.40	4	0.00701
A0A097PIJ6	53.05	11.00	4	0.00457
A0A097PIJ9	32.58	7.40	4	0.00698
A0A0A0MQX7	26.72	8.50	4	0.00028
A0A0A0MRA3	0.22	3.40	4	0.00000
A0A0A0MRG2	7.66	4.17	6	0.00017
A0A0A0MS15	16.80	2.00	4	0.00067
A0A0A0MTS7	0.15	2.96	4	0.00000
A0A0B4J1T9	31.02	3.44	4	0.00121
A0A0B4J1V6	15.95	2.09	4	0.00050
A0A0B4J1Z4	21.79	2.15	6	0.00061
A0A0B4J259	12.78	2.00	4	0.00029
A0A0C4DGL8	13.63	4.50	4	0.00048
A0A0F7TAV4	32.68	2.94	4	0.00117
A0A0G2JIE7	16.25	5.80	4	0.00047
A0A0G2JL69	16.03	9.10	6	0.00045
A0A0H3W5J2	57.64	16.20	4	0.00911
A0M8Q6	54.87	5.83	6	0.00692
A0N5G3	37.59	4.22	4	0.00223
A2J1N8	18.90	2.00	4	0.00063
A2J422	7.00	2.00	4	0.00400
A2J423	7.37	2.00	4	0.00408
A2KBB9	29.14	5.24	4	0.00060
A2KBC0	29.14	5.24	4	0.00060
A2KBC2	29.14	5.24	4	0.00060
A2KBC3	29.14	5.24	4	0.00060
A2KBC4	29.73	5.61	4	0.00060
A2KBC5	29.14	5.24	4	0.00060
A2KBC7	29.14	5.24	4	0.00060
A2KBC8	29.14	5.24	4	0.00060
A2KUC4	13.91	2.43	4	0.00097
A2MYE1	29.42	2.31	6	0.00074
A2MYE2	29.42	2.31	6	0.00074
A2NB45	32.70	3.30	8	0.00157
A4UGR9	1.53	2.50	4	0.00002
A5YM51	3.30	2.00	4	0.00005
A6XMH4	28.40	4.27	6	0.00094

A8K2I0	18.59	11.82	4	0.00123
A8K3L0	24.77	13.50	4	0.00046
A8K5T0	30.16	27.56	6	0.00063
A8K7Q1	11.63	4.47	4	0.00013
A8K9M5	8.97	3.80	6	0.00019
B0YJC4	7.63	3.18	6	0.00015
B0ZBE2	27.43	9.61	6	0.00063
B1AHL2	15.94	7.63	6	0.00022
B2R4C5	23.80	2.85	6	0.00036
B2R6W1	15.93	8.95	8	0.00048
B2R9F2	17.85	5.29	4	0.00024
B2RCU8	10.82	2.38	4	0.00015
B3GQS7	4.40	2.00	6	0.00011
B3KRK8	8.08	3.18	6	0.00016
B3KS70	59.38	13.12	4	0.00154
B4DID6	44.12	11.81	4	0.00217
B4DJT9	7.66	4.17	6	0.00017
B4DMX2	7.33	3.00	4	0.00019
B4DPR2	83.94	111.50	6	0.06588
B4DQM1	7.42	4.17	6	0.00016
B4DR57	51.03	8.25	4	0.00131
B4DRB7	7.10	3.54	4	0.00009
B4DRF2	22.76	13.00	4	0.00045
B4DRR0	22.22	13.83	4	0.00130
B4DSV9	3.13	2.57	4	0.00011
B4DV48	16.03	9.10	6	0.00045
B4DZ36	3.54	3.00	4	0.00013
B4DZX0	8.86	3.20	4	0.00014
B4E1B3	28.56	9.61	6	0.00065
B6EDE2	18.03	2.00	4	0.00046
B6V6K7	13.35	3.86	4	0.00017
B7Z1F8	47.34	15.07	4	0.00128
B7Z4F6	4.50	2.00	6	0.00012
B7Z544	28.44	21.00	4	0.00081
B7Z597	5.46	2.00	6	0.00012
B7Z5E7	5.96	2.00	6	0.00013
C9J0D1	24.32	2.00	6	0.00065
C9JC84	20.21	7.80	4	0.00075
C9JEU5	20.94	7.80	4	0.00077
C9JV77	42.95	12.57	4	0.00194
D3DP13	32.36	7.86	4	0.00137
D3DSM4	3.42	2.94	6	0.00006
D3DSM5	3.02	2.94	6	0.00005
E7ETN3	32.18	36.88	4	0.00103
E9PBC5	7.33	3.00	4	0.00019
E9PR44	51.88	11.50	6	0.01400
F5GY80	10.00	3.80	6	0.00021
F5H5G1	12.69	2.38	4	0.00017
F5H7G1	9.83	3.80	6	0.00021
F8VV32	16.83	2.00	4	0.00038
G3GAU4	20.60	2.00	4	0.00186
G5E968	11.05	2.00	4	0.00033

H0Y300	27.58	12.88	4	0.00038
H0Y3H6	16.20	5.80	4	0.00047
H0Y897	2.89	2.57	4	0.00010
H3BLU2	10.58	2.38	4	0.00014
H6VRF9	25.64	16.73	4	0.00032
H6VRG3	15.08	8.27	4	0.00032
H7BXV5	4.22	2.40	6	0.00011
H7BYX6	6.70	3.55	4	0.00007
H7C0V9	10.72	4.67	4	0.00014
I0B0K5	2.23	2.00	4	0.00002
I0B0K6	2.08	2.00	4	0.00002
I0B0K7	1.98	2.00	4	0.00001
I0B0K8	1.30	2.00	4	0.00001
I6L894	1.25	2.00	4	0.00001
J3QLC9	18.63	5.75	4	0.00046
J3QR68	30.16	12.88	4	0.00042
K7ERE3	8.30	3.00	4	0.00019
K7ES70	12.58	2.50	4	0.00014
P00918	39.91	9.17	4	0.00054
P01019	27.43	9.61	6	0.00063
P01617	32.25	3.06	8	0.00157
P02489	53.57	13.67	6	0.01211
P02511	51.58	11.50	6	0.01392
P02533	20.43	8.87	4	0.00107
P02538	21.09	13.83	4	0.00123
P02743	11.55	2.69	4	0.00044
P04114	4.90	17.14	4	0.00005
P04259	14.52	10.12	6	0.00084
P05813	31.98	7.40	4	0.00685
P06314	20.74	4.00	4	0.00086
P06681	17.09	10.27	4	0.00054
P08670	8.75	4.00	6	0.00014
P08779	19.70	8.14	4	0.00075
P0C0S5	23.19	2.00	6	0.00062
P10809	5.44	2.00	6	0.00011
P12036	1.85	2.50	4	0.00007
P12883	1.60	2.50	4	0.00005
P13533	1.60	2.50	4	0.00005
P13591	7.71	5.31	4	0.00006
P13646	10.39	4.43	4	0.00017
P20396	18.66	2.11	4	0.00041
P20930	1.30	2.00	4	0.00001
P22914	35.08	6.60	6	0.00741
P24593	15.68	2.82	6	0.00060
P32119	56.90	11.83	4	0.00131
P36980	19.78	3.69	4	0.00033
P39060	2.62	2.94	6	0.00004
P48668	17.34	11.38	4	0.00119
P53673	53.05	11.00	4	0.00457
P53674	36.48	9.00	4	0.00651
P55083	13.84	2.50	4	0.00015
P61626	23.80	2.85	6	0.00036

P81605	27.72	3.10	4	0.00081
Q01484	1.25	2.00	4	0.00001
Q02818	11.63	4.47	4	0.00013
Q06830	34.14	5.09	4	0.00045
Q06AH7	80.48	95.21	6	0.01120
Q08629	22.95	8.50	4	0.00024
Q0ZCH4	43.99	4.36	4	0.00950
Q0ZCH6	15.30	2.00	4	0.00061
Q0ZCJ2	41.54	4.36	4	0.00897
Q12841	14.06	3.65	4	0.00039
Q13449	10.82	2.38	4	0.00015
Q14118	5.11	2.80	4	0.00011
Q14204	1.20	2.25	4	0.00001
Q17R60	6.48	4.29	6	0.00014
Q53GX6	11.63	4.47	4	0.00013
Q53GY3	25.90	9.12	4	0.00070
Q53GZ8	16.35	9.40	4	0.00054
Q53HP3	16.05	9.67	6	0.00043
Q53HU8	8.75	4.00	6	0.00014
Q59EV3	1.95	2.50	4	0.00006
Q59FB2	10.43	3.67	4	0.00014
Q5JP69	15.74	9.27	6	0.00043
Q5NV88	38.10	3.17	4	0.00111
Q5SR54	12.37	7.14	4	0.00028
Q65ZC8	7.00	2.00	4	0.00058
Q6EMK4	5.43	2.21	4	0.00006
Q6EZE9	23.37	2.00	4	0.00095
Q6N041	24.97	9.25	6	0.00118
Q6N095	35.74	23.78	4	0.00561
Q6PJF2	47.41	14.67	4	0.01096
Q6PJG0	41.94	11.71	4	0.00479
Q6S8J3	4.40	4.14	4	0.00025
Q6ZW64	44.19	17.60	4	0.00176
Q71UI9	23.19	2.00	6	0.00062
Q86T07	9.20	2.00	4	0.00027
Q86YZ3	2.82	4.93	4	0.00007
Q8IZZ5	15.28	7.09	6	0.00031
Q8NG19	11.09	2.40	6	0.00030
Q8TE63	14.23	2.60	4	0.00117
Q96DK0	43.63	17.22	4	0.00175
Q99972	10.67	4.59	4	0.00012
Q9BU40	9.55	3.50	4	0.00013
Q9GZL9	82.35	18.18	4	0.00524
Q9UL85	26.86	2.63	6	0.00090
S6B286	38.26	9.00	4	0.00456
S6BAN6	42.42	10.40	4	0.00470
V9HW12	56.90	11.83	4	0.00131
V9HW21	39.91	9.17	4	0.00054
V9HW27	51.58	11.50	6	0.01392
V9HWE1	8.75	4.00	6	0.00014
V9HWP0	11.55	2.69	4	0.00044

a n=sample size

b NSAF=Normalized spectral abundance factor

supplementary table S2: 86 proteins identified only in IVC group

Uniprot ID	sequence coverage(%)	sequence count	n[a]	mean NSAF[b]
P01042	32.16	20.72	5	0.001370864
A0A0A0MS11	19.00	2.00	5	0.000637962
A0A0B4J1V5	16.68	2.10	5	0.000619388
A2J1M2	18.40	2.00	5	0.000739473
A2J1M3	20.36	2.10	5	0.000754879
A2JA19	22.53	2.33	5	0.000442968
B4DNT5	11.56	2.90	5	0.000342119
B4DVE1	14.58	7.22	5	0.000259104
B4E1C2	32.16	20.72	5	0.001370864
B4E1E1	14.97	6.00	5	0.000227741
Q5CZ94	38.99	10.70	5	0.002171826
Q5NV90	35.34	3.69	5	0.001705882
Q6PIK1	41.71	11.25	5	0.00284313
Q8N5F4	42.76	10.71	5	0.004629495
Q8NCL6	44.54	17.14	5	0.001932507
Q9UL86	46.14	4.29	5	0.000735982
Q9UNU2	52.94	15.40	5	0.001143534
S6B2A6	22.54	8.30	5	0.001770215
U5LGU2	10.90	2.90	5	0.000323437
U5LGW0	10.90	2.90	5	0.000323437
U5LH96	10.90	2.90	5	0.000323437
U5LI90	10.90	2.90	5	0.000323437
U5LIC5	10.90	2.90	5	0.000323437
U5LIG7	10.90	2.90	5	0.000323437
U5LIH6	10.90	2.90	5	0.000323437
U5LII8	10.90	2.90	5	0.000323437
U5LKC6	10.90	2.90	5	0.000323437
U5LKG0	10.90	2.90	5	0.000323437
U5LKM6	10.90	2.90	5	0.000323437
U6A3P2	99.73	9.78	5	0.021600361
O75326	16.95	9.67	5	0.000374211
Q08380	14.27	7.22	5	0.000253789
Q4G0P3	0.73	2.50	5	1.91391E-05
P00915	51.66	12.92	6	0.001320625
Q09666	0.82	2.56	6	1.16306E-05
P01598	28.24	2.65	6	0.000766631
P01700	19.00	2.92	6	0.000708331
A0A024R1G8	8.49	3.00	6	0.000216953
A0A024R1Q4	8.82	3.00	6	0.000225674
A0A075B6N7	43.77	10.86	6	0.001767259
A5PL32	7.98	3.00	6	0.000204598
B2R9E5	8.49	3.00	6	0.000216953
B3KUE5	7.31	2.38	6	8.24437E-05
B3KXC3	11.03	2.23	6	0.000245187
O14791	8.82	3.00	6	0.000225674
B4E1N5	10.35	3.00	6	0.000264951
B7WNR0	85.22	104.77	6	0.061524122
B7Z539	24.41	12.75	6	0.000875543
E9PKY7	17.71	2.23	6	0.00039359

E9PRK8	21.34	2.23	6	0.000472308
H0Y789	27.31	2.50	6	0.000385302
P17948	4.73	11.33	6	0.000284996
L7RSL3	4.73	11.33	6	0.000284996
Q1WWL5	8.13	3.00	6	0.00020888
Q29RW7	8.13	3.00	6	0.00020888
Q2KHQ6	8.82	3.00	6	0.000225674
Q53H91	7.29	2.20	6	8.57883E-05
Q6NZ44	11.03	2.23	6	0.000245187
Q6P2J1	39.00	9.67	6	0.00249194
Q6VFQ6	98.80	7.67	6	0.008757382
U5LH46	12.73	3.17	6	0.000356159
U5LHF6	12.73	3.17	6	0.000356159
U5LKR8	12.73	3.17	6	0.000356159
V9HWE3	51.66	12.92	6	0.001320625
P55058	7.60	2.38	6	8.57883E-05
Q8N475	11.26	8.50	6	0.000209334
P00739	22.98	8.67	6	0.000658368
P02794	14.56	2.75	7	0.00028636
P04003	21.81	9.33	7	0.000334726
A0A024R525	14.56	2.75	7	0.00028636
A0A024RD88	5.71	10.44	7	0.001372374
A0A075B6J0	35.20	2.64	7	0.000572552
A2J1M8	24.71	2.36	7	0.000915726
B4DVQ0	15.20	4.11	7	0.000483955
Q5NV69	41.50	2.64	7	0.000675611
Q6NS36	11.49	2.75	7	0.000225879
Q8TD27	15.48	2.50	7	0.0003176
O43866	17.65	4.50	7	0.000252825
P35968	5.71	10.44	7	0.001372374
A0A087WYR4	42.14	11.26	8	0.002879086
Q68CN4	33.63	15.80	8	0.003502381
Q8IWM0	34.81	3.13	8	0.000884123
S6BGE0	29.95	10.57	8	0.004281338
P02745	11.95	2.39	9	0.000269131
A0A024RAG6	11.95	2.39	9	0.000269131
Q96JD0	34.25	3.35	9	0.001572333

a n=sample size

b NSAF=Normalized spectral abundance factor

Supplementary table S3: Differentially expressed proteins among the 450 protein detected in both PDR and IVC group

Uniprot ID	Protein Name	sequence coverage(%)	sequence count	n[a]			NSAF[b]			*P* value
				PDR	IVC	control	PDR	IVC	control	
P02652	Apolipoprotein A-II（APOA2）	63.03	9.17	8	8	9	* 0.005264	0.004816	0.004155	0.0465
P02675	Fibrinogen beta chain（FGB）	48.27	24.04	6	8	7	* 0.002382	0.002974	0.001064	0.0071
B7Z5Q2	highly similar to Ceruloplasmin	60.02	62.60	4	5	5	0.003117	* 0.002461	0.003442	0.0086
P15692	Vascular endothelial growth factor A（VEGFA）	27.80	3.00	8	7	8	* 0.006122	0.002493	0.002225	0.0001
Q6ZVX0	highly similar to Protein Tro alpha1 H,myeloma	36.12	14.54	8	7	9	* 0.002824	0.001579	0.001538	0.0095
V9HWF6	Alpha-1-acid glycoprotein(AGP)	43.01	11.90	8	8	9	* 0.003785	0.002265	0.003116	0.0027
P59665	Neutrophil defensin 1(DEFA1)	23.37	2.00	4	5	0	* 0.000953	0.000496	-	0.0235
P59666	Neutrophil defensin 3(DEFA3)	23.37	2.00	4	5	0	* 0.000953	0.000496	-	0.0235
Q9UBP4	Dickkopf-related protein 3(DKK3)	47.55	13.56	4	5	9	* 0.002554	0.001352	0.001797	0.0201
P19652	Alpha-1-acid glycoprotein 2(AGP2)	37.22	8.00	8	8	9	* 0.002628	0.001620	0.002348	0.0063
Q96IY4	Carboxypeptidase B2（CPB2）	14.98	4.06	6	5	3	* 0.000225	0.000296	-	0.0111
Q6MZV6	DKFZp686L19235	37.78	15.08	8	8	8	* 0.001964	0.001655	0.001670	0.0346
P02679	Fibrinogen gamma chain（FGG）	42.92	20.50	8	8	7	0.001594	* 0.001978	0.000863	0.0225
A0A0B4J1X5	Protein IGHV3-74	18.65	2.16	6	7	9	* 0.005985	0.000495	0.000677	0.0448
Q96K68	highly similar to Homo sapiens SNC73 protein (SNC73) mI	44.02	16.62	8	8	9	* 0.002265	0.001986	0.001724	0.0489
Q9NPP6	Immunoglobulin heavy chain variant	42.33	14.56	6	7	9	* 0.002494	0.002069	0.001782	0.0115
Q9UL83	Myosin-reactive immunoglobulin light chain variable region	38.81	3.31	8	7	7	* 0.001579	0.000741	0.000947	0.0120
Total										17

a n=valid sample size

b NSAF=normalized spectral abundance factor, which means the relative abundances of protein

"*"=has a statistically significant difference with other groups

"-"=not identificated by LC-MS/MS
